# Sepsis and the Heart: In the Quest for Noninvasive Pressure-Volume Loops at the Bedside

**DOI:** 10.1097/CCE.0000000000001328

**Published:** 2025-09-30

**Authors:** Pedro D. Salinas, Jon Barnett, Siddharth Dugar

**Affiliations:** 1 Aurora Critical Care Services, Aurora Sinai/Aurora St. Luke’s Medical Centers, University of Wisconsin School of Medicine and Public Health, Milwaukee, WI.; 2 Department of Critical Care Medicine, Integrated Hospital Care Institute, Cleveland Clinic, Cleveland, OH.

**Keywords:** left ventricle ejection fraction, myocardial work, Sepsis, septic cardiomyopathy, ventricular elastance, ventriculo-arterial coupling

## Abstract

**BACKGROUND:**

Sepsis-induced cardiomyopathy (SICM) is prevalent yet remains difficult to diagnose using conventional echocardiography primarily due to its dependence on loading conditions, the dynamic nature of sepsis, and varied cardiovascular phenotypes. Recent advancements in noninvasive myocardial work (MW) analysis, particularly through left ventricle pressure-strain loop (LV PSL), offer a promising strategy for evaluating myocardial function by combining strain imaging with blood pressure data. This technique may address the limitations inherent in traditional measures such as ejection fraction, which can be influenced by fluctuating hemodynamics in sepsis and may not accurately reflect underlying myocardial function.

**CASE SUMMARY:**

This report presents three cases wherein patients exhibited either preserved or only mildly reduced left ventricular systolic function based on ejection fraction (LVEF), but were found to have diminished MW indices, including global work index, global constructive work, and global work efficiency along with low flow by left ventricle outflow-tract.

**CONCLUSIONS:**

Relying solely on LVEF for diagnosing SICM is problematic due to numerous confounding variables. MW parameters constitute innovative, noninvasive echocardiographic indicators that have demonstrated value across a spectrum of cardiac disorders. Although these parameters appear promising as bedside assessment tools, their application within the context of sepsis warrants further investigation.

**KEY POINTS**

**Question**: Which bedside echocardiographic parameters are useful for assessing cardiac function in patients with sepsis-induced cardiomyopathy (SICM)?

**Findings**: myocardial work (MW) parameters may demonstrate abnormalities indicative of SICM, even when left ventricular ejection fraction appears preserved.

**Meaning**: MW parameters represent promising, noninvasive echocardiographic measures; however, their clinical significance remains to be clearly established at this time.

## BACKGROUND

Sepsis, a syndrome of dysregulated response to infection leading to organ failure, remains associated with significant morbidity and mortality (1, 2). Cardiac dysfunction, known as sepsis-induced cardiomyopathy (SICM), is common in sepsis (3, 4). The prevalence of SICM is between 40% and 60%; however, the lack of a standard definition, different cardiac phenotypes in sepsis (5), and lack of a standardized test at the bedside that reflects true myocardial dysfunction affect the accuracy of the estimated prevalence (5–9). Invasive PVL analysis with a high-fidelity catheter is the gold standard for understanding ventriculo-arterial coupling (VAC) using pressure-volume (PVL) relationships during the cardiac cycle. It provides information on the inherent myocardial contractility and myocardial energetics under varying loading conditions (10). Unfortunately, clinical use of invasive PVL analysis is prohibitive and impractical outside research settings. Instead, a conventional parameter, like left ventricular ejection fraction (LVEF), is usually used to identify and define SICM. Some limitations of LVEF are lack of sensitivity and dependence on loading conditions, often appearing preserved in patients with vasoplegia and unmasked once systemic vascular resistance is corrected (6). Using the Chen method, a high frequency of ventriculo-arterial uncoupling in sepsis has been reported (11). Chen et al (12) described this echocardiographic parameter-based algorithm as a noninvasive single-beat alternative to assess end-systolic ventricular elastance in various cardiovascular conditions, comparing it against invasive pressure-volume loop (PVL) measurements. Although this evaluation can be performed at the bedside, it remains time-consuming and requires multiple precise Doppler measurements making it cumbersome for rapid serial monitoring as a bedside tool (13). Speckle tracking echocardiography-derived left ventricle global longitudinal strain (LV GLS) measures the shortening of myocardial fiber during systolic contraction indexed to diastolic muscle fiber length expressed as percentage (14). The negative percentage portrays a more robust systolic function.

**Figure 1.**
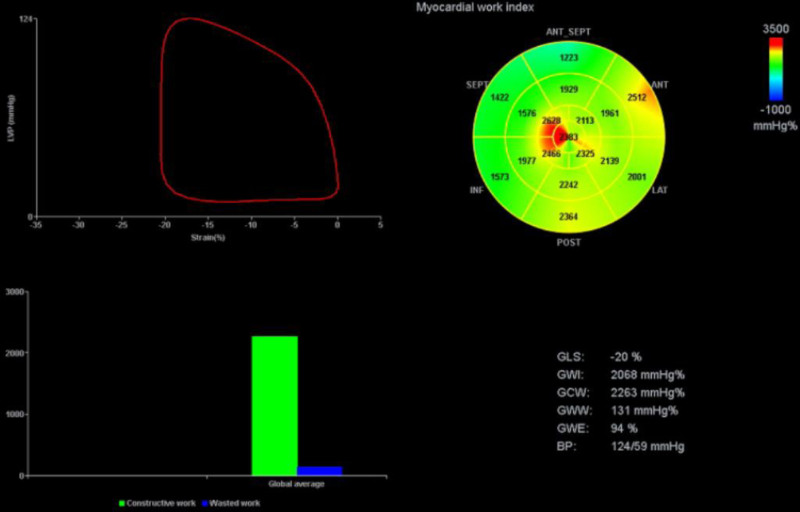
Myocardial work (MW) and pressure-strain loop screen display. Left ventricle pressure-strain loop on the upper left. MW index *bulls-eye graph* on the right upper corner and *graphical bar* depiction of global construction work (GCW) and global wasted work (GWW). GLS = global longitudinal strain, GWE = global work efficiency, GWI = global work index.

Despite increased sensitivity of LV GLS, it is impacted by loading conditions (15). The left ventricle pressure-strain loop (LV PSL) is a noninvasive echocardiographic parameter that has been validated against invasive PVLs in both animal and human models, and provides a novel method of quantifying MW, with potential advantages over conventional LV GLS by correction of loading conditions (16). LV PSL integrates GLS with blood pressure as a surrogate for left ventricular pressure and afterload. This combined approach enables a more precise evaluation of intrinsic LV function and may help identify subclinical myocardial dysfunction. A proprietary GE Healthcare software aligns BP to key cardiac event timings, namely isovolumic contraction, systolic ejection, and isovolumic relaxation (IVR), as identified through echocardiographic assessment of valvular opening and closure events. This provides MW parameters that can help distinguish true myocardial dysfunction from compensated cardiac function, as well as insights into myocardial energetics and wasted work (**Table 1**, **Fig. 1**)(17–19). Herein, we describe three cases of hospitalized septic patients where LV PSL significantly added to our understanding of cardiac function. The cases were selected from a large dataset with identifying information modified. Approval from the institutional review board (IRB00113154) was secured accompanied by a waiver of consent.

**TABLE 1.** Myocardial Work Parameter Definitions and Reference Range (17–19)

Parameter	Abbreviation	Description	Key Components	Reference Range
Global work index	GWI	Total myocardial work during mechanical systole(including IVC&IVR). Includes positive and negative work (abnormal) during cardiac systole.	Area under the pressure-strain loop; including isovolumetric contraction, ejection phase, and isovolumetric relaxation. From MV closure to MV opening	2062 ± 269 mm Hg% (M), 2155 ± 275 mm Hg% (F)
Global constructive work	GCW	Represents the energy consumed by the myocardium that contributes to cardiac output.	Shortening during isovolumetric contraction (IVC) and ejection pluslengthening during isovolumic relaxation (IVR)	2229 ± 275 mm Hg% (M), 2283 ± 286 mm Hg% (F)
Global wasted work	GWW	Represents the myocardial work that does not contribute to cardiac output.	Lengthening during isovolumetric contraction and ejection, plus shortening during isovolumetric relaxation	60 (42–84) mm Hg% (M), 68 (49–93) mm Hg% (F)
Global work efficiency	GWE	Reflects the percentage of total myocardial work that contributes effectively to cardiac output. Calculated as GCW/(GCW + GWW).	Ratio of constructive to total work (GCW and GWW).	97%

F = female, M = male, MV, mitral valve.

### Case 1

**Figure 2.**
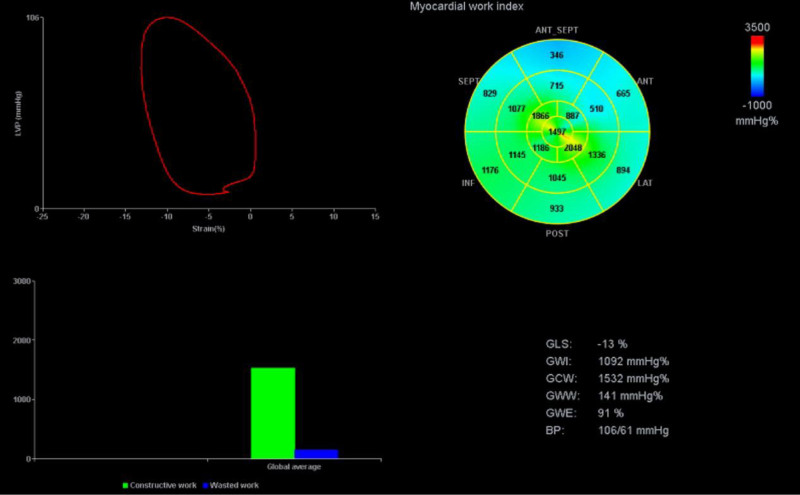
Left ventricle pressure-strain loop (PSL) on upper left. Myocardial work (MW) index *bulls-eye graph* on right upper corner and *graphical bar* depiction of global construction work (GCW) and global wasted work (GWW). The rightward deviation with decreased PSL area signifies decreased myocardial mechanical energy with increased wasted work. GLS = global longitudinal strain, GWE = global work efficiency, GWI = global work index.

A 35-year-old man with respiratory distress, was diagnosed with streptococcal pneumonia, and required mechanical ventilation. Upon admission, his vital signs were hazard ratio (HR) 91 beats/min, BP 119/67 mm Hg, and he was afebrile. He was on dobutamine at 2.5 µg/kg/min and vasopressin at 0.03 units/min. His transthoracic echocardiogram (TTE) revealed an LVEF of 45%, indicating mild left ventricular systolic dysfunction, along with a low left ventricular outflow tract velocity-time integral (LVOT VTI) of 13.5 cm. LV PSL showed (**Fig. 2**) that the global work index (GWI) was markedly reduced to 1092 mm Hg (Table 1), whereas global constructive work (GCW) was 1532 mm Hg%, global wasted work (GWW) was 141 mm Hg%, and global work efficiency (GWE) was 91%, all of which were abnormal. The patient recovered with antibiotics and supportive care. This case highlights abnormal MW parameters despite normal LV systolic function as assessed by conventional LVEF.

### Case 2

A 25-year-old woman with a history of hypertrophic cardiomyopathy, right ventricular dysfunction with severe tricuspid regurgitation, end-stage renal disease was admitted with confusion, fever, and found to have meningitis. Her vital signs were HR 80 beats/min, BP 85/59 mm Hg, and she was afebrile. No vasoactive medications were used. A TTE showed preserved LVEF of 56%. LVOT VTI was reduced to 15.5 cm. MW parameters (**Supplemental Fig. 1**, https://links.lww.com/CCX/B559) showed a GWI 788 mm Hg%, GCW 1064 mm Hg%, GWW 110 mm Hg%, GWE 90%; all consistent with severe global LV systolic dysfunction despite pseudomonal LVEF. The patient recovered uneventfully.

### Case 3

A 40-year-old woman with a history of IV drug use presented with sepsis and was found to have tricuspid valve endocarditis and septic lung emboli. Her vital signs were HR 107 beats/min, BP 108/69 mm Hg, and she was afebrile. She was placed on dobutamine during her ICU stay. A TTE showed a large tricuspid valve vegetation, mild right ventricle (RV) cavity enlargement with normal RV systolic function, and mild pulmonary hypertension with an right ventricular systolic pressure of 40 mm Hg. Her LVEF was preserved with 56% and low LVOT VTI of 12.6 cm. MW parameters were obtained (**Supplemental Fig. 2** https://links.lww.com/CCX/B559): GWI 886 mm Hg%, GWE 93%, GCW 1489 mm Hg%, and GWW 108 mm Hg%. These findings indicated severe myocardial systolic dysfunction on MW parameters despite a pseudo-normal LVEF. She underwent percutaneous endovascular aspiration of the tricuspid valve vegetation and was eventually discharged home.

## DISCUSSION

SICM, particularly impaired left ventricular systolic function as indicated by reduced LVEF, is highly prevalent, with widely variable reported rates largely due to rapidly fluctuating loading conditions after interventions such as fluid resuscitation and vasoactive therapies influencing assessed LVEF. In the cases we present, there is a notable discrepancy between traditional cardiovascular performance parameters, such as LVEF, and stroke volume surrogates, such as LVOT VTI. This discrepancy further highlights the poor correlation between preserved LVEF and low flow. Therefore, in critically ill patients, flow measurements hold greater relevance (20, 21). Even though PVL analysis can clarify true myocardial contractility and myocardial consumption in different loading conditions, its use is limited to research settings (22). The MW analysis produces an LV PSL along with the following parameters: GWI, GCW, GWW, and GWE (23). These parameters provide information regarding MW and energetics similar to invasive pressure-volume analysis (24). Russell et al (16), showed a close correlation of areas between (*r* = 0.96) the PVL and PSL, and those loops for comparison are illustrated in **Supplemental Figure 3** (https://links.lww.com/CCX/B559). However, MW software necessitates the integration of LV GLS images from apical four, three, and two chamber views. Despite the automation in the analysis, the apical cardiac images must be of high quality, which poses a significant limitation for widespread application in the critically ill population (25).

The influence of loading conditions on cardiac mechanics and energetics by MW parameters is an active area of research. For instance, preserved LVEF, LV GLS with elevated GWI, GWW, and decreased GWE may be seen in conditions such as systemic hypertension reflecting increased wasted work and decreased work efficiency despite preserved systolic function. In contrast, reduced LV GLS accompanied by increased GWI and GCW can be observed in pressure or volume overload states such as aortic stenosis or aortic regurgitation. Finally, a pattern of both reduced LV GLS and decreased MW is typically associated with heart failure with reduced ejection fraction, indicating decompensated myocardial dysfunction (23).

Only one prospective study has been published evaluating MW parameters in patients with sepsis, with survivors demonstrating higher GWE compared with non-survivors at day 3. GWI on day 1 was marginally different between survivors and non-survivors (1587 vs. 1165 mm Hg%, *p* = 0.05). LVEF was not different between survivors and non-survivors (26). We have also reported significantly decreased GWI in our cohort of 179 patients presenting with sepsis and preserved LVEF (27).

The cases discussed would be categorized as normal LV systolic function using LVEF. However, decreased GWI, GCW, and GWE were noted in all three patients. In two cases, the use of inotropic support was initiated due to low LVOT VTI and MW parameters consistent with poor flow and ventriculo-arterial uncoupling. These cases highlight the need to identify and distinguish different abnormalities in myocardial performance, such as decreased myocardial function, increased wasted work, and abnormalities in VAC that could potentially have diagnostic and therapeutic relevance (22, 28, 29). However, other questions must be addressed, including the early identification of sepsis-related cardiomyopathy with more sensitive parameters of MW that correct for loading effects in cardiac function. Guarracino et al (30) showed this heterogenous response in a group of patients with sepsis, where volume expansion improved hemodynamics and LVEF in all patients. Still, norepinephrine failed to improve hemodynamics in up to 40% of patients with persistent hypertension. Using the noninvasive bedside VAC, a significant increase in the arterial elastance/end-systolic elastance (Ea:Ees) ratio was shown in non-responders (uncoupling of VAC) compared with responders (1.80 ± 0.62 vs. 1.09 ± 0.17, *p* < 0.05). Notably, those patients who had increased cardiac output response to norepinephrine also had increased ventricular elastance and decreased Ea:Ees ratio, implying improved VAC.

SICM, as measured by low LVEF and LV GLS, is influenced by variable preload and afterload, making it difficult to accurately assess true myocardial dysfunction in sepsis. MW parameters are new noninvasive echocardiographic tools studied in other cardiovascular diseases, but their role in sepsis is not well established. More research is needed to determine whether MW can reliably identify cardiac dysfunction and guide treatment in sepsis.

## Supplementary Material
