# Supplementary material for: Sepsis and the Heart: In the Quest for Noninvasive Pressure-Volume Loops at the Bedside
Source: Crit Care Explor. 2025 Sep 30;7(10):e1328. doi: 10.1097/CCE.0000000000001328 (PMC12487935; doi:10.1097/CCE.0000000000001328)
Supplement: Supplementary file 1 [file cc9-7-e1328-s001.pdf]

Supplemental Digital Content

Sepsis and the Heart: In the Quest for Non-Invasive Pressure-Volume Loops at the Bedside

Table of Contents

Supplemental Figure 1: Case 2 – Myocardial Work & Pressure Strain Loop (Page 2)

Supplemental Figure 2: Case 3 – Myocardial Work & Pressure Strain Loop (Page 3)

Supplemental Figure 3: Pressure-Volume vs. Pressure-Strain Loop (Page 4)

Myocardial Work and Pressure-Strain Loop

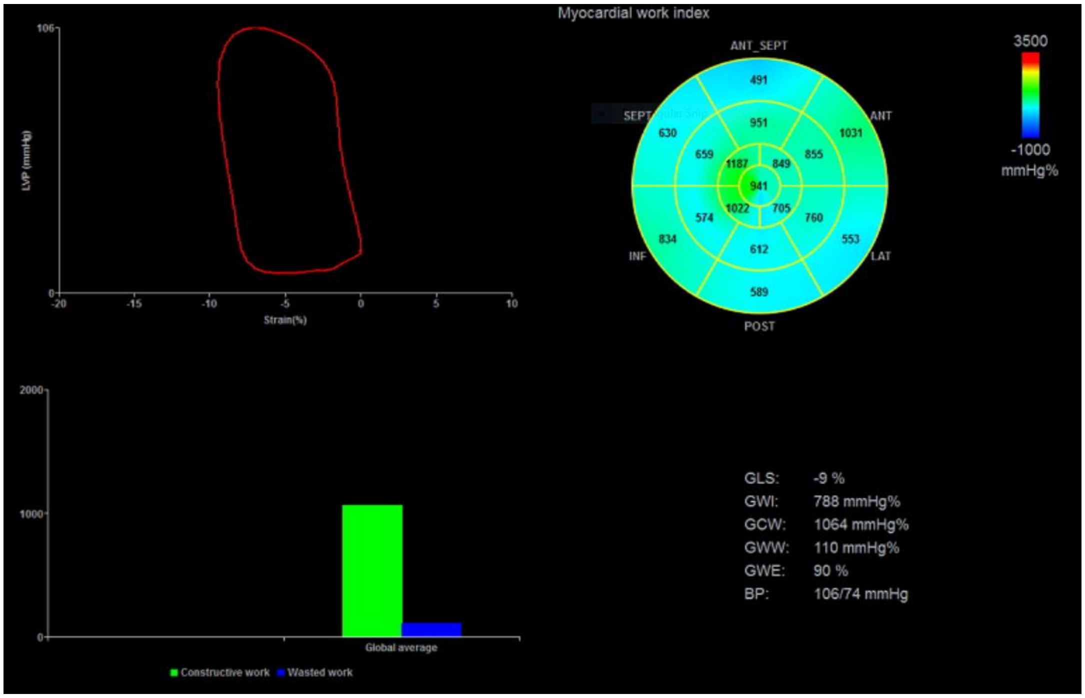

Supplemental Figure 1.Left ventricle pressure-strain loop (LV PSL) on upper left with right and upward shift. Myocardial Work (MW) Index bulls eye graph on the upper right corner with very low values and graphical bar depiction of decreased global construction work (GCW) and increased global wasted work (GWW).

Myocardial Work and Pressure-Strain Loop

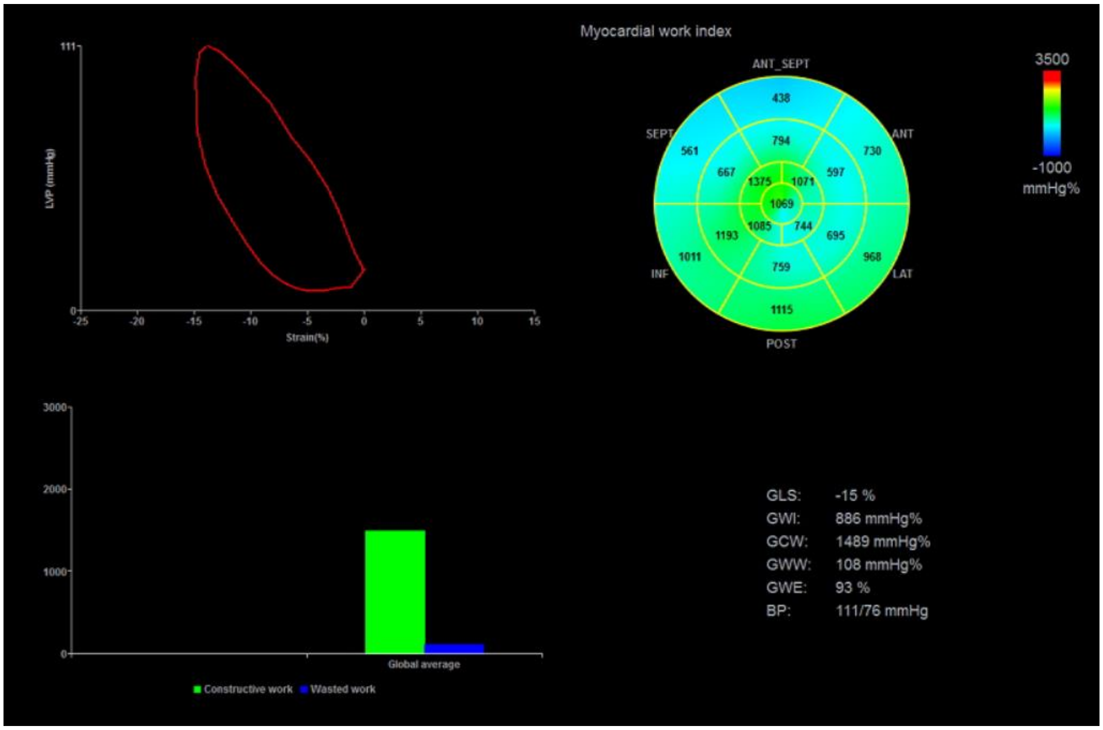

Supplemental Figure 2. Left ventricle pressure-strain loop (LV PSL) on upper left corner demonstrating right shift. Myocardial Work (MW) index bulls eye graph on the upper right corner with low values and graphical bar depiction of decreased global work index (GWI), global construction work (GCW), increased global wasted work (GWW) and decreased global work efficiency (GWE).

Pressure-Volume vs Pressure-Strain Loop

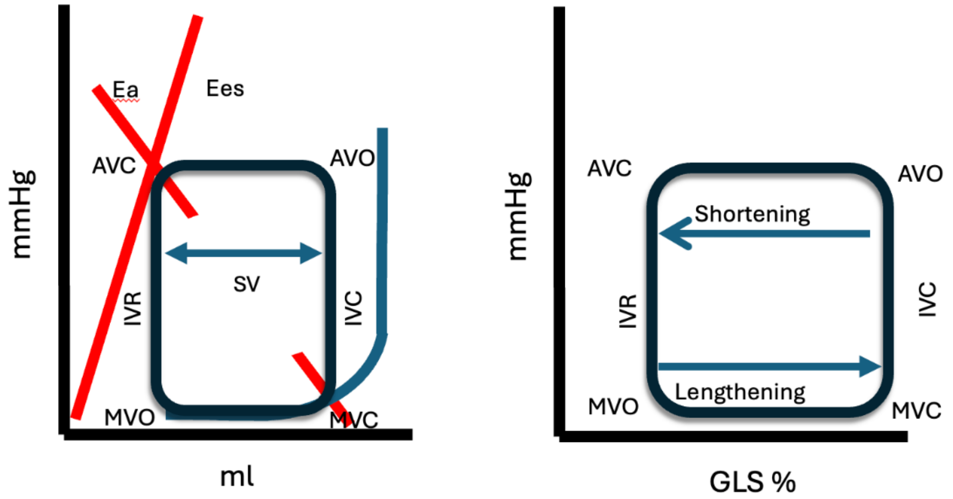

Supplemental Figure 3. Depiction of classical pressure-volume loop. X-axis represent volume in ml and Y-axis pressure in mmHg. Mitral valve opening (MVO) marks the initiation of diastole and mitral valve closure (MVC) ends diastole. The period of isovolumetric contraction (IVC) starts with MVC and ends with aortic valve opening (AVO). Between AVO and aortic valve closure (AVC) the pressure remains minimally changed but ejection takes place and accounts for the stroke volume (SV). The end-systolic slope of the pressure-volume loop represents ventricular elastance (Ees) an that line represents the myocardial contractility at different pressure-volume points. The intersection of Ea/Ees represents the interaction of arterial and ventricular elastance and its effects in stroke volume. On the right side of the figure a pressure-strain loop is depicted, X-axis represent GLS% instead of volume, and Y-axis pressure in mmHg. Both loops retain close correlation in different conditions.
